# Low-temperature CO oxidation on multicomponent gold based catalysts

**DOI:** 10.3389/fchem.2013.00012

**Published:** 2013-09-06

**Authors:** Tomás Ramírez Reina, Svetlana Ivanova, Miguel A. Centeno, José A. Odriozola

**Affiliations:** Departamento de Química Inorgánica e Instituto de Ciencia de Materiales de Sevilla, Centro mixto CSIC-Universidad de SevillaSevilla, Spain

**Keywords:** gold catalysts, CO oxidation, cerium oxide, iron oxide, mixed oxides

## Abstract

In this work the development of gold catalysts, essentially based on γ-alumina with small superficial fraction of Ce-Fe mixed oxides as support for the low temperature CO oxidation is proposed. Characterization results obtained by means of TEM, OSC, XPS, UV-Vis spectroscopy and H_2_-TPR are employed to correlate the activity data with the catalysts composition. The bare γ-alumina supported gold catalyst demonstrates the poorest activity within the series. The addition of CeO_2_ or FeO_X_ improves the catalytic performance, especially observed for the CeO_2_-FeO_x_ mixed oxide doped samples. This enhanced CO oxidation activity was related to the Ce-Fe interaction producing materials with promoted redox properties and therefore oxidation activity.

## Introduction

Gold has been long discarded from catalytic applications due to the inert nature of massive gold. However, it is now about 30 years since the original breakthroughs of Hutchings (1985) and Haruta et al. (1987) demonstrating that gold can be an excellent catalyst when supported on metal oxides in a highly dispersed state. Since this discovery, intensive research has been devoted to the determination of the reactions types that can be efficiently catalyzed by gold nanoparticles, to the increase of the stability of the gold nanoparticles avoiding their sintering, to understand the role of the support in the catalytic activity and to demonstrate the similarities and distinctive properties of gold catalysis with respect to other noble metals (Corma and García, 2008) etc.

Within the properties that make gold unique as a catalyst, the ability of gold to catalyze reactions at ambient and sub-ambient temperature is one of the most important. The later opens up totally new application opportunities creating an entirely new field of low temperature catalysis with characteristics not previously found (Corti et al., 2007). For example gold catalysts are commonly deactivated by nanoparticles agglomeration, however, this sintering phenomena does not occur at sub ambient and room temperature. Furthermore, the poisoning of gold catalysts via carbonate formation due to the interaction of the support with atmospheric CO_2_ can be avoided by storing the catalysts in a closed container at room temperature (Corti et al., 2007). The catalyst can preserve its activity under these conditions for 2 years (Cortie et al., 2006).

The oxidation of CO over gold catalysts is one of the most studied reactions in the last 20 years due to the high activity of gold catalysts for this reaction at ambient and sub-ambient temperature. Nowadays, a number of possible applications for catalysts able to perform CO oxidation at ambient temperature (as for example gold based catalysts) appear. Within the most extended application it should be mention: carbon dioxide lasers, gas sensors, respirators for protecting firefighters and miners from the CO poisoning, air-cleaning devices and many environmental issues.

The activity of gold-based catalysts toward CO oxidation is known to be sensitive to (1) the preparation method, (2) the nature of the supports, (3) the size of the gold particles, (4) the pre-treatment conditions, and (5) to the gold—support interactions (Trim, 2005). In particular, the choice of the support is of crucial relevance. Currently gold supported on a reducible transition metal oxide exhibit a significantly enhanced activity for CO oxidation compared to gold supported on non-reducible materials due to their ability to supply reactive oxygen. Au supported on CeO_2_ and to lower extent on CeO_2_-MO_x_ mixed oxides have been broadly studied (Gluhoi et al., 2003; Laguna et al., 2010). The relevance of these cerium based supports is mainly based on their high oxygen storage capacity (OSC), which is directly correlated to the creation, stabilization and diffusion of oxygen vacancies, especially on the oxide surface, due to the reversible redox behavior of the Ce^4+^/Ce^3+^couple (Veccheti et al., 2011).

The formation of oxygen vacancies in a CeO_2_-MO_x_ supportis directly related to the formation of Ce-M solid solution. The dopant ions with electronegativity and ionic radius close to those of cerium cation are consider the most appropriate modifiers of structural and chemical properties of ceria. The presence of these dopants can provoke structural distortions such as ceria lattice contraction thus favoring the formation of oxygen vacancies. Among the transition metal cations, Fe^3+^ has been successfully employed as a CeO_2_ doping agent for CO oxidation reactions. The best oxidation activity usually is reported when Ce-Fe solid solution is formed and attributed to the increase of the ceria oxygen vacancies population (Laguna et al., 2011). Nevertheless, in some cases, above a certain amount, iron is not completely incorporated to the ceria network but segregates and it can play a role of an electronic modifier allowing a better dispersion of the added noble metal (Reddy et al., 2010).

Not at last place, the recently emerged new trend in the scientific community have to be taken into consideration, namely the need of lowering the rare earth metal compounds use and dependence, which leads us to consider the probability to substitute the bulk rare oxide support with a small superficial layer of it deposited on an “inert” oxide, such as alumina.

For all discussed above, the main goal of this work is the development of an efficient series of gold catalysts for low temperature CO oxidation using CeO_2_-FeO_x_ mixed oxides supported on Al_2_O_3_ as a support. The evaluation of the catalytic performance induced by the presence of certain amounts of iron in the catalyst formulation is another objective of this paper since the addition of iron accounts for the high performance of these systems and their viability in any possible environmental application.

## Experimental details

### Catalyst preparation

#### Synthesis of the supports

In a typical preparation no matter ternary or quaternary systems, the desired amount of Ce(NO_3_)_3_× 6H_2_O and Fe(NO_3_)_3_× 9H_2_O (Aldrich) were impregnated on γ-alumina powder (Sasol). The impregnation was carried out in 50 mL of ethanol, evaporated till obtaining of a dry solid in rotary vapor at reduced pressure and temperature of 50°C. The obtained solid was treated with NH_3_ (Aldrich) solution 10 mol · L^−1^ during 30 min in order to assure the full conversion of the nitrates to hydroxides precursors. The support was then filtered, dried and calcined at 500°C for 4 h.

The initial precursor quantities (CeO_2_ or FeO_x_) are calculated to be 15 wt.% of the final solid with FeO_x_ to CeO_2_ promotion varying from 0 to 3 wt.%. The samples are labeled as CeFeX/Al, where X indicates the theoretical FeO_x_ (always calculated as Fe_2_O_3_) loading.

#### Gold deposition

The gold deposition was performed by the direct anionic exchange method (DAE) (Ivanova et al., 2004). Aqueous solutions of the gold precursor HAuCl_4_ (Johnson Matthey) 2 × 10^−4^ M and support sieved in between 100 and 200 μm were used in order to obtain a final Au loading of 2 wt.%. The solution was heated to 70°C and aged 20 min. After that the solution was cooled down to 40°C and 50 mL of NH_3_ (30% Aldrich) ware added. The slurry was then filtered, washed with water, dried in an oven at 100°C overnight and calcined in air at 350°C for 4 h.

***Caution/safety note*.** The contact of ammonia with gold solution can result in the formation of gold ammonia complexes (“fulminating gold”) which are explosive. Using this washing procedure may not be dangerous as the gold complexes are strongly attached to the support by DAE. Similar to the supports, in the adopted nomenclature the real gold loadings are omitted for simplification and the FeO_x_ contents are expressed as the theoretical wt.% loading in the catalysts. For example, the Au/CeFe0.5/Al solid contains 2 wt.%. Au loading over 15 wt.% of Ce-Fe mixed oxide on Al_2_O_3_ support in which the FeO_x_ loading is 0.5 wt.% approximately.

### Characterization techniques

The chemical composition of the samples was determined by X-Ray microfluorescence spectrometry (XRMF) in an EDAX Eagle III spectrophotometer with a rhodium source of radiation.

High-Resolution Transmission Electron Microscopy (HRTEM) and High-Angle Annular Dark Field-Scanning Transmission Electron Microscopy (HAADF-STEM) images were recorded on a JEOL2010F instrument. The spatial resolution at Scherzer defocus conditions in HRTEM mode is 0.19 nm, while the HAADF-STEM studies were performed using an electron probe of 0.5 nm of diameter and a diffraction camera length of 10 cm. The average size of Au particles and its distributions were estimated by counting about 70 Au particles.

The Temperature Programmed Reduction (TPR) experiments were carried out in a conventional quartz reactor connected to a thermal conductivity detector (TCD). The reactive gas stream, 5% H_2_ in Ar (Air Liquide) was passed through a 50 mg of sample with a flow rate of 50 ml.min^−1^ and the temperature rose at 10°C · min^−1^ from room temperature to 900°C. A molecular sieve 13× was used to retain the H_2_O produced during the reduction and/or CO_2_ which could be desorbed from the solid surface.

For the Oxygen Storage Complete Capacity (OSCC) 100 mg of catalyst was loaded into a U-shaped quartz reactor and the temperature was raised in a He flow (50 mL/min) until 350°C. Then, 10 O_2_ pulses of 1 mL were injected every 2 min. After that, the sample is submitted to 10 CO pulses of 1 mL each (every 2 min). The sample is then degassed during 10 min in a He flow and subjected to a new series of oxidizing pulses (10 O_2_ pulses) and then exposed to five alternating pulses (CO–O_2_–CO–O_2_–CO–O_2_). The OSC is determined by the amount of CO_2_ formed after the first CO pulse of the alternated ones. This method is based on the one presented by Duprez et al. (Kacimi et al., 1993; Royer and Duprez, 2011). The gas composition at the exit of the reactor was analyzed by a mass spectrometer PFEIFFER Vacuum PrismaPlus controlled by the program Quadera®.

The UV-Vis spectra were recorded on a Varian® spectrometer model Cary 100, equipped with an integrating sphere and using BaSO_4_ as reference. All the spectra were collected in a diffuse reflectance mode and transformed to a magnitude proportional to the extinction coefficient through the Kubelka-Munk function F(α).

X-ray photoelectron spectroscopy (XPS) analyses were performed on a Leybold-Hereus LHS-10/20 photoelectron spectrometer equipped with multichannel analyzer EA200. The sample powders pressed in small stainless steel troughs of 4 mm diameter were placed on a ceramic carousel. The pressure in the analysis chamber was around 5 × 10^−9^ Torr. The analyzed area was ~1.4 mm^2^ and the pass energy was set at 150 eV. Spectra were obtained by using Mg Kα_1,2_ radiation (1253.6 eV). The binding energies were calculated with respect to the C(C, H) component of the C 1 s peak fixed at 284.8 eV. Data treatment was performed with the CasaXPS program (Casa Software Ltd., UK). The atomic fractions were calculated using peak areas normalized on the basis of acquisition parameters and sensitivity factors provided by the manufacturer.

### Catalytic activity set-up

The activity measurements were carried out in a U-shape glass flow reactor at atmospheric pressure. The catalysts were pretreated in a 30 mL min^−1^ activation flow of 21% O_2_ balanced in He (from room temperature to 350°C, 5°C min^−1^). After the activation, a reactive flow 3.4% CO (Air Liquide, 99.997%), and 21% O_2_ (Air Liquide, 99.999%) balanced by helium, was passed through the reactor at room temperature. The gas total flow was 42 mL min^−1^ and the quantitative analysis was carried out with a Blazers OmnistarBentchop mass spectrometer. The catalysts were tested in the reaction flow at room temperature until reached the steady state. Then, the systems were heated to 350°C at 5°C · min^−1^.

When necessary, the same reaction was carried out at temperatures below 0°C. The reactor, loaded with a fresh and activated sample, was immersed into a cooling bath composed by liquid N_2_ and acetone. Once the temperature stabilized, the reactive flow described above was flushed through the reactor. The temperature was increased slowly to the room temperature and the quantitative analysis of the reactives and products was carried out on a Blazers OmnistarBentchop mass spectrometer calibrated using gas mixtures of CO and CO_2_ in helium.

## Results and discussion

### Chemical composition: bulk and surface

The chemical composition of the prepared solids is presented in Table 1. Bulk composition of the supports seems to be close to the intended values. All the Ce-Fe mixed systems presented similar amounts of cerium oxide (~15 wt.%) and iron oxide increased from 0.62 wt.% in the CeFe0.5 sample till 2.42 wt.% in the CeFe3 sample. Gold deposition provokes a slight decrease in both cerium and iron oxide loadings. This phenomena could be related to the highly basic media (NH_3_, 30% v/v) employed during the gold deposition. It seems that a part of the support oxides is dissolved during the process affecting the catalysts composition. Regarding gold loadings, the experimental values neighboring 2 wt.% are obtained, excepting the Au/Al_2_O_3_ catalyst for which a 40% of gold loss is observed. It was reported elsewhere (Ivanova et al., 2006) that always 30–32% of gold loss is observed when the DAE method assisted by NH_3_ is applied for the preparation of Au supported on alumina.

**Table 1:** **Chemical composition of the prepared solids**.

**Sample**	**CeO_2_(wt.%)**	**FeO_x_(wt.%)**	**Al_2_O_3_(wt.%)**	**Au(wt.%)**	**Ce/Fe**	**Au/Ce**	**S_BET_ (m^2^/g)**
Al_2_O_3_	–	–	100	–	–	–	202
Au/Al_2_O_3_	–	–	98.8	1.2	–	–	217
Ce/Al	11.1 (25.7)	–	88.9	–	–	–	186
Au/CeAl	10.8 (20.1)	–	87.5	1.7 (0.4)	–	0.030	197
Fe/Al	–	14.7 (17.9)	85.3	–	–	–	200
Au/FeAl	–	13.6 (17.7)	84.5	1.9 (0.9)	–	–	202
CeFe0.5/Al	17.1 (24.7)	0.6 (1.0)	82.2	–	11.2	–	174
Au/CeFe0.5/Al	14.1 (19.1)	0.4 (8.1)	83.6	1.83 (0.8)	1.7	0.035	213
CeFe1/Al	16.8 (20.2)	0.9 (2.5)	82.2	–	21.2	–	195
Au/CeFe1/Al	16.7 (18.8)	0.9 (1.2)	80.1	2.3 (1.5)	21.1	0.048	171
CeFe1.5/Al	15.9 (21.1)	1.3 (3.8)	82.7	–	7.5	–	183
Au/CeFe1.5/Al	12.9 (34.5)	1.1 (3.3)	84.0	2.1 (1.9)	20.27	0.049	195
CeFe2/Al	16.2 (24.4)	2.1 (4.6)	81.7	–	20.48	–	175
Au/CeFe2/Al	14.9 (22.6)	1.7 (4.0)	81.2	2.2 (1.7)	22.54	0.046	184
CeFe3/Al	14.4 (26.6)	2.4 (5.9)	83.2	–	26.50	–	190
Au/CeFe3/Al	13.7 (24.2)	2.4 (3.7)	81.1	2.0 (1.7)	27.51	0.035	195

*Bulk composition outside the parenthesis obtained by X-Ray Micro Fluorescence (XRMF). Surface composition inside the parenthesis obtained by XPS*.

For the surface composition the atomic ratios of all present components from the XPS analysis have been used to calculate the corresponding mass ratios for sake of comparison in between the two methods of characterization. However, the Ce/Fe and Au/Ce atomic ratios were also included in the Table 1. Analyzing the results several points should be considered; the surface layer composition and its relation to the bulk composition. Concerning the surface layer composition, the XPS results show that approximately 20–25 wt.% of the mixed oxide is presented on the catalysts surface on which the gold particles have been deposited. Nevertheless, the surface and bulk compositions significantly differ. An enrichment of cerium and iron oxides in the solids surface is observed compared to the bulk composition, confirming the successful deposition of the Ce-Fe mixed oxide on the alumina surface. All the solids present between 19 and 25 wt.% of superficial ceria loading except the Au/CeFe1.5/Al sample where the cerium oxide content on the surface was remarkably higher (34.5 wt.%). The increase of the iron oxide layer is proportional to that of ceria one excepting the Au/Fe0.5/Al sample, where the iron oxide surface concentration is higher than expected, as confirmed also by the lowest Ce/Fe atomic ratio. As for the gold compositions, a general decrease of gold amount was obtained compared to the bulk values. As a detail, this decrease on the available gold on the surface seems to be less important within the Ce-Fe mixed oxides. The formation of the Ce/Fe solid solution within this series of samples have been already discussed in details in reference (Reina et al., 2013) and confirmed for the majority of the samples, being the CeFe2/Al the limiting value above which the FeOx phase segregation occurs. Considering this solid solution formation an increase of the amount of ceria oxygen vacancies is expected with the increase of the extent of solid solution formation. As proposed by Laguna et al. (2010) these structural defects may act as nucleation centers for gold deposition thus improving the gold dispersion linearly with the concentration of defects. The later agrees with the fact that Au/CeFe1.5/Al is the system that admits the higher amount of gold on the surface since this system presents the highest concentration of defects on its surface and a strong gold-support contact (Reina et al., 2013). Concerning the textural properties, the catalysts are mesoporous materials with specific surface areas of around 200 m^2^ · g^−1^ governed by the presence of the primary γ-Al_2_O_3_ support. A slight increase of the specific surface area of the final solids after gold deposition is observed. This effect has been reported earlier (Konya et al., 2003) and attributed to the increase of the pore volume caused by the presence of gold nanoparticles inside the pore structure.

### X-ray photoelectron spectroscopy spectra (XPS)

**Figure 1:**
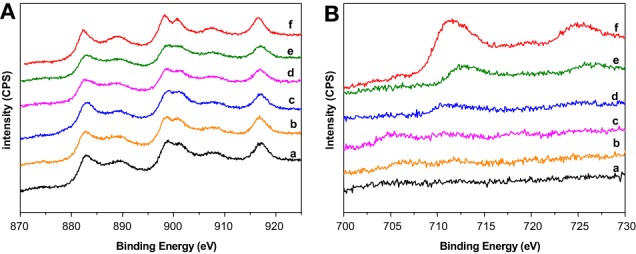
**XPS spectra of the prepared supports. (A)** Ce3d region: (a) Ce/Al, (b) CeFe0.5/Al, (c) CeFe1/Al, (d) CeFe1.5/Al, (e) CeFe2/Al, (f) CeFe3/Al; **(B)** Fe2p región: (a) CeFe0.5/Al, (b) CeFe1/Al, (c) CeFe1.5/Al, (d) CeFe2/Al, (e) CeFe3/Al, (f) Fe/Al.

Selected XPS spectra of the prepared supports are presented in Figure 1. Ce3d spectra are shown in Figure 1A. Six peaks corresponding to three pairs of spin–orbit doublets can be identified. The complex shape of the cerium 3d spectra arises from the multiplet effect such as hybridization of Ce 4f orbitals with O 2p levels and final state (Le Normand et al., 1988) and is characteristic of CeO_2_. However, the presence of Ce^3+^ must not be discarded. Slight but important differences can be extracted from the spectra, if the attention is focused on Ce^3+^. Currently, the main Ce^3+^ peaks appear at about 885 and 905 eV. In our mixed systems, especially from CeFe1/Al to CeFe2/Al, these peaks can be noticed overlapped with the Ce^4+^ ones. The presence of Ce^3+^ could be linked to the existence of oxygen vacancies which appear along the support series due to the formation of Ce-Fe solid solution. The presence of Ce^3+^ in the CeFe3/Al sample is less notorious according to the shape of the spectra. This system marks the limit where Ce-Fe solid solution no longer occurs in good agreement with the discussion published in reference Reina et al. (2013).

Iron oxide, Fe 2p spectra is presented in Figure 1B. Fe/Al exhibited two XPS peaks attributed to Fe 2p_3/2_ and Fe 2p_1/2._ The Fe 2p_3/2_ is narrower and stronger and with greater area than Fe 2p_1/2_ caused by the spin-orbit (j-j) coupling effect; Fe 2p_3/2_ has degeneracy of four states whilst Fe 2p_1/2_ has only two states. The obtained binding energies of Fe 2p_3/2_ and Fe 2p_1/2_ for the Fe/Al sample are 711.4 ± 0.2 and 724.96 ± 0.2 eV, respectively, in good agreement with the data obtained by Yamashita and Hayes (2008) for a Fe_2_O_3_ standard. In addition, the Fe 2p_3/2_ peak in Fe_2_O_3_ must have a satellite located at ~8 eV higher than the main Fe 2p_3/2_ peak. In our sample, this satellite can be intended at 719.2 ± 0.2 eV. The small amount of iron in the rest of the solids limits the resolution of the spectra, however, for the CeFe3/Al and CeFe2/Al the same profile that the one observed for Fe/Al was obtained with a slight shift that may account to the Ce-Fe interaction.

**Figure 2:**
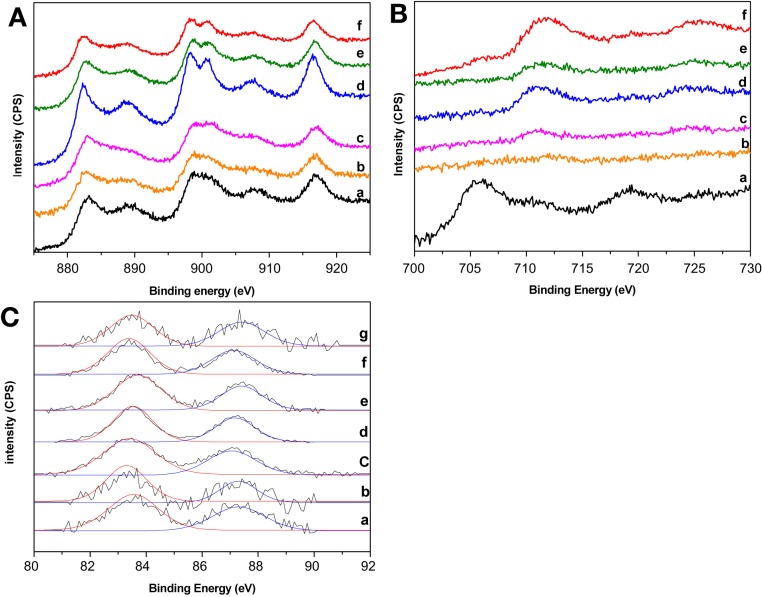
**XPS spectra of the prepared gold catalysts. (A)** Ce3d region: (a) Au/Ce/Al, (b) Au/CeFe0.5/Al, (c) Au/CeFe1/Al, (d) Au/CeFe1.5/Al, (e) Au/CeFe2/Al, (f) Au/CeFe3/Al; **(B)** Fe2p región: (a) Au/CeFe0.5/Al, (b) Au/CeFe1/Al, (c) Au/CeFe1.5/Al, (d) Au/CeFe2/Al, (e) Au/CeFe3/Al, (f) Au/Fe/Al; **(C)** Au 4f region (a) Au/Ce/Al, (b) Au/CeFe0.5/Al, (c) Au/CeFe1/Al, (d) Au/CeFe1.5/Al, (e) Au/CeFe2/Al, (f) Au/CeFe3/Al, (g) Au/Fe/Al.

XPS spectra of the prepared gold catalysts are presented in Figure 2. Not remarkable changes in the Ce 3d spectra were found after gold deposition (Figure 2A) indicating that there is no change in the electronic density of ceria atoms which remains mainly as Ce^4+^. Nevertheless, it should be pointed out that Au/CeFe1.5/Al sample did modify its profile in some extension toward a better defined Ce^4+^ which agrees with the increased of the superficial CeO_2_ presented in Table 1. The later could also be related to the fact that gold tend to nucleate on the ceria oxygen vacancies (associated with Ce^3+^) thus covering the small portion of Ce^3+^ on the catalyst surface. As for the Fe 2p spectra (Figure 2B), the inclusion of gold did not significantly modify the profiles, except from the Au/CeFe0.5/Al sample where gold deposition seems to unearth Fe but in a different oxidation state since the peaks position are far from the rest of the series. In this sample the binding energies of Fe 2p_3/2_ and Fe 2p_1/2_ are 706.3 ± 0.2 and 719.6 ± 0.2 eV, respectively, close to the energies obtained for a Fe_0.94_O standard in reference Yamashita and Hayes (2008) confirming the change of the iron oxidation state in this particular case, which also results in some incertitude in the calculation of the Ce/Fe atomic ratio. Finally Au 4f spectra are reported in Figure 2C. In this region each gold species shows two peaks Au 4f_7/2_ at 83.4 ± 0.2 eV and Au 4f_5/2_ at 87.2 ± 0.2 eV characteristic of metallic gold (Haruta et al., 1993). A weak shift on the peak positions was observed. This shift may indicate differences on the Au-Ce interaction as proposed by Rodriguez et al. (Ma et al., 2007) and in good agreement with the properties derived from the support composition in terms of Ce-Fe interaction and the presence of ceria electronic defects that could alter gold electronic density. Each peak has been successfully fitted with a Guassian-Lorenztian curve with one component supporting the metallic character of gold in this series of catalysts. However, this interaction is not the only factor to consider for explaining the shift since a change in the gold particle size should be also taken into account. Furthermore, the assumption that gold is present on its metallic form is quite reasonable considering that samples have been calcined at 350°C. It was in fact published that at calcination temperatures higher than 300°C gold is present just as Au^0^ (Park and Lee, 1999).

### TEM

**Figure 3:**
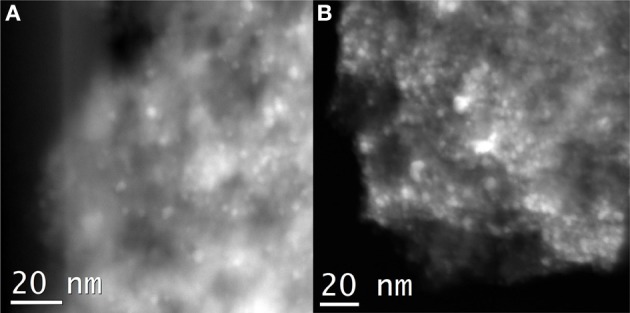
**Selected TEM images of the prepared gold catalysts. (A)** Au/CeAl; **(B)** Au/CeFe2/Al.

Selected TEM images of the gold catalysts are shown in Figure 3. The white spots correspond to the presence of heavier elements such as Ce, Ce-Fe, and/or Au supported on alumina. No clear differences in dispersion, size and shape are evidenced among the catalysts. However, we must take into account that the high atomic weight of cerium atoms in the samples makes the detection of gold particles difficult due to the low mass and diffraction contrast. Although, it should be pointed out that in all samples the gold particles are regularly distributed on the whole support and the particle size ranges from 3 to 5 nm as reported in (Reina et al., 2013). As for example, TEM measurements for Au/Ce/Al found 3.8 ± 0.5 nm gold average size and 4 ± 0.2 nm for the Au/CeFe2/Al sample which make us to consider 4 nm average gold particle size for all calculation, e.g., dispersion, reaction rate etc. It should be pointed, however, that even if this slight differences do not influence the normalization of the catalytic activity can significantly contribute to the shift in the XPS gold peaks as pointed above.

### UV-vis spectroscopy

**Figure 4:**
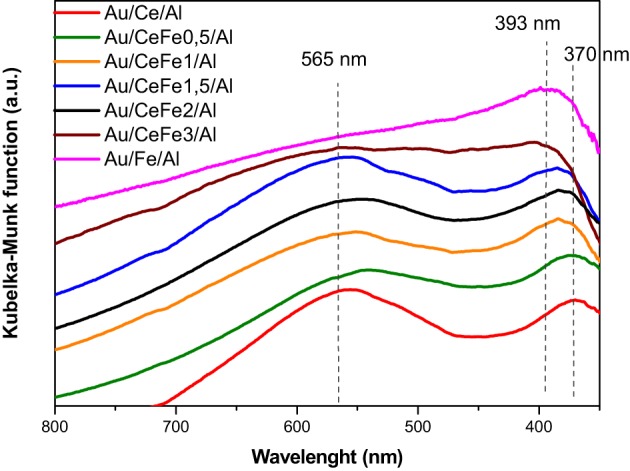
**UV-Vis spectra of the prepared gold catalysts**.

UV-vis spectra of the prepared gold catalysts are presented in Figure 4. All the Au/Ce-Fe_x_/Al solids show a broad band situated at about 370 nm in the UV region accounting for the ceria UV-Vis features. This band is due to the charge transfer from 2p valence band of O^2−^ to 4f band of Ce^4+^ (Centeno et al., 2002). As a relevant phenomena, a widening and shifting of the ceria absorption edge is observed evidencing Ce-Fe interaction. It should be mentioned that the shift of the ceria absorption edge occurs till 2 wt.% of Fe content since the Au/CeFe3/Al sample did not show this band. A weaker Ce-Fe interaction could be expected for this solid. In addition this sample and the Au/Fe/Al sample exhibited a broad absorption at 393 nm. This band was attributed by Reddy and co-workers to O → Fe^3+^ ligand-to-metal charge transfer of isolated Fe ions in tetrahedral and octahedral coordination (Reddy et al., 2010).

Furthermore, a broad absorption band was found in the range 500–570 nm for most of the samples. This contribution is assigned to the well-known plasmon resonance of gold nanoparticles. For the Au/Fe/Al sample the gold contribution is masked by the extended absorption of iron oxide in the visible region ascribed to d-d transitions ^6^A_1g_ → ^4^T_1g_ and ^6^A_1g_ → ^4^T_2g_ in α-Fe_2_O_3_ (Reddy et al., 2010). This contribution could be envisaged also for the Au/CeFe3/Al system for which the higher iron concentration suggests oxide segregation (Reina et al., 2013).

### TPR

Redox properties are of especial relevance when oxidation reactions are considered. An approximation to the redox behavior of the gold catalysts was obtained by the TPR-H_2_ experiments. Figure 4 shows the TPR-H_2_ profiles of the prepared solids.

**Figure 5:**
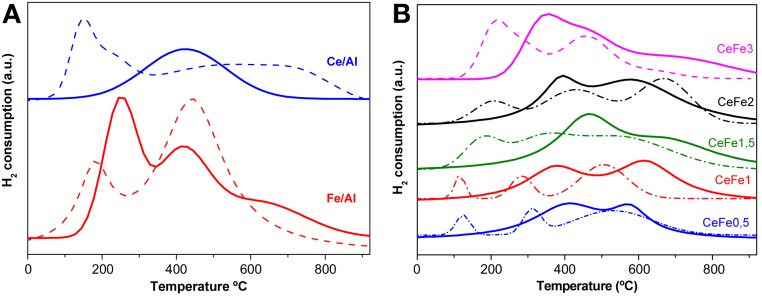
**TPR-H_2_ profiles of the supports (full lines) and their corresponding gold catalysts (dashed lines). (A)** Binary systems. **(B)** Ce-Fe ternary systems.

The Fe_2_O_3_-Al_2_O_3_ support (Figure 5A) presents 3 overlapping zones of reduction corresponding to the two steps of the reduction from Fe^3+^ to Fe^2+^ and to Fe^0^ of the surface and bulk iron oxide. Wimmers et al. (1986) studied the reduction of Fe_2_O_3_ and proposed a reduction in two steps Fe_2_O_3_→ Fe_3_O_4_→ Fe, with no formation of FeO. For the same oxide, other authors proposed a three steps reduction process considering FeO formation dealing with: Fe_2_O_3_→Fe_3_O_4_ at about 400°C, Fe_3_O_4_ → FeO at about 600°C and finally FeO → Fe at higher temperatures (Boccuzzi et al., 1999). The later is in good agreement with our profile. The CeO_2_-Al_2_O_3_ (Figure 5A) presents only one reduction zone at around 495°C attributed to the Ce^4+^ to Ce^3+^ reduction of the superficial ceria. As for the Ce-Fe mixed supports (Figure 5B) two zone of reduction can be clearly seen in the temperature range (300–600°C). The two reduction peaks involve both iron oxide and cerium oxide reduction process that are not possible to separate. The main different was observed in the first reduction zone that becomes more intense and shifts to lower temperatures (except in the CeFe1.5 sample) with the iron load in the sample pointing the Ce-Fe synergetic effect. This peak could be due to the first reduction step of Fe^3+^ species (Laguna et al., 2011). However, the simultaneous reduction of Ce^4+^ and Fe^3+^ cannot be discarded. Finally the CeFe3/Al presented a shoulder at around 700°C as was observed in the Fe/Al sample reinforcing that iron segregation occurs in this solid as it was mentioned in UV-Vis data discussion.

Concerning to the gold catalysts (Figures 5A ternary system and 5B quaternary system), as expected the presence of gold decreases the temperature of reduction by facilitating the mobility of the H_2_ molecule on the surface of the solid. It was reported earlier (Jacobs et al., 2004) a shift in the reduction peaks related to the surface oxygen of CeO_2_ in the presence of gold at much lower temperatures Because of the later, Au/Ce/Al sample shows two reduction zones while the Ce/Al solid shows only one. The low temperature reduction (centered at 150°C) zone is assigned to the noble metal promoted ceria surface reduction and the high temperature reduction process is ascribed to the ceria bulk reduction. An overlapping of the latest reduction steps in the Au/Fe/Al system was observed. In the quaternary Au/CeFe/Al systems, the addition of gold provoked the apparition of three peaks instead of the two original ones. In fact the last reduction peak must be attributed once again to the bulk ceria reduction. Currently the amount of consumed hydrogen was higher for the Ce-Fe mixed systems pointing that this quaternary systems possess enhanced redox properties which generally leads to a better catalytic behavior in CO oxidation reactions.

### OSCC and OSC

**Figure 6:**
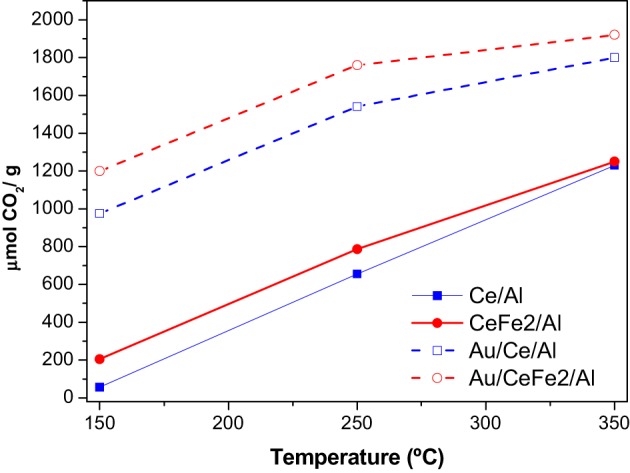
**OSCC and OSC of the Ce/Al and CeFe2/Al samples (full symbol and lines) and Au containing analogues (empty symbols dashed lines)**.

For further understanding of the promoter effect of iron addition to the ceria redox properties, OSCC and OSC measurements of Ce/Al and CeFe2/Al samples and their corresponding gold analogs were carried out. The OSCC provides information about the maximum reducibility of the samples while the OSC informs about the most reactive and most available oxygen atoms. The OSCC measurements expressed as the sum of the μmolCO_2_ formed for all the CO pulses contacted with the solid, as a function of the temperature is presented in Figure 6. As a general trend the increase of the temperature increases the OSCC value more pronounced for the supports than for the catalysts. The Fe containing samples exhibited superior OSCC in the whole studied temperature range. This result indicates that the inclusion of iron in the catalyst formula enhanced the oxygen mobility. In order to find out the number of oxygen layers involved in the process the measurements of the OSC was carried out. For the OSC theoretical calculations, the number of surface oxygen atoms and the BET area of each sample (Table 1) were considered. More precisely it was considered that (1) only oxygen atoms bonded to the cerium ones participate in the oxygen storage process; (2) the surface is considered homogeneous (3) only one of the four oxygen atoms is involved in the storage (CeO_2_ → Ce_2_O_3_ + “O”) (4) null gold metal contribution to the reduction, e.g., the gold metal could not be reoxidized after the calcinations of the samples.

The results are presented in Table 2. For the supports, similar to the OSCC higher the temperature higher the number of oxygen layers involved in the reduction of the surface—effect enhanced by the presence of iron. However, almost no accessible oxygen for the immediate exchange was found at 150°C. The addition of gold changes dramatically the oxygen mobility in the whole temperature range, especially at the lowest temperature, where the oxygen availability increases around 30 times. Nevertheless, for the rest of the temperatures a maximum seems to be reached indicating that above 250°C the number of oxygen layers remains constant.

**Table 2:** **Oxygen storage capacity (OSC) and number of oxygen layers (NL) as a function of the temperature**.

**Sample**	**OSC_150°C_**	**OSC_250°C_**	**OSC_350°C_**	**NL_150°C_**	**NL_250°C_**	**NL_350°C_**
Ce/Al	15	209	479	0.01	0.22	0.5
CeFe2	21	275	492	0.02	0.32	0.69
Au/Ce/Al	368	650	671	0.33	0.58	0.60
Au/CeFe2/Al	360	662	678	0.38	0.73	0.75

## Catalytic activity

### CO oxidation

**Figure 7:**
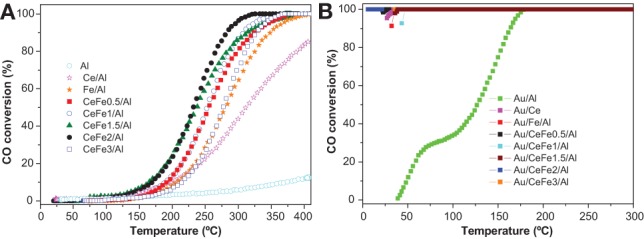
**CO oxidation light-off curves of the (A) supports; (B) gold catalysts**.

Figure 7A shows the light-off CO oxidation curves for the prepared supports. All the ternary systems based on Ce-Fe mixed oxide supported on Al_2_O_3_ exhibited a superior catalytic activity compared to the binary systems Fe/Al or Ce/Al evidencing the beneficial effect of introducing iron oxide to ceria. It has been recently reported that a noticeable increase of the catalytic performance in CO oxidation for a ceria-alumina commercial support can be achieved by employing iron oxide as a doping agent (Reina et al., 2012). It was demonstrated that the incorporation of iron species into ceria lattice creates structural sites with high electronic density acting as preferential place where CO molecules oxidized. The activity of the supports corresponds to the following order: CeFe2/Al › CeFe1.5/Al › CeFe1/Al › CeFe0.5/Al › CeFe3/Al › Fe/Al › Ce/Al › Al. The later agrees with the enhanced electronic properties exhibited for the CeFe2/Al sample as demonstrated by the OSC experiments.

Concerning gold catalysts, all of them (except Au/Al and Au/Fe/Al) reached total conversion at room temperature (Figure 7B). These results evidences the different behavior between gold supported on reducible oxides such as iron and cerium oxide and gold supported on non-reducible oxides such as alumina. The high activity of gold-ceria catalysts may be attributed to the presence of reactive oxygen as observed by OSC measurements for the ceria containing samples which suggests a support assisted CO oxidation via Mars van Krevelen mechanism. Higher the OSC higher the oxygen mobility and better the catalytic behavior toward CO oxidation.

**Figure 8:**
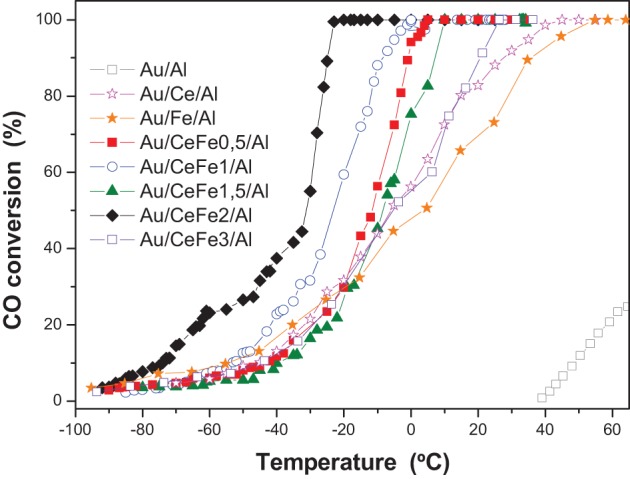
**Sub-ambient CO oxidation light-off curves of the gold catalysts**.

In order to discern better the catalytic behavior, a sub-ambient CO oxidation study was carried out. Figure 8 shows the activity test at low temperature for the gold catalysts. The specific reaction rates at 0°C and 25°C and turnover frequencies (TOF), using the dispersion of Au atom exposure to reactants flow, are calculated and presented in Table 3. For the dispersion a mathematical model consisting of estimating the average size of the gold nanoparticles as a function of the selected geometry and gold atomic radii was employed and directly used (Ivanova et al., 2006). The TOF then is the normalization of the reaction rate to the dispersion values expressed in s^−1^.

**Table 3:** **Low temperature CO oxidation reaction rates and TOF values for the gold catalysts at 0°C and 25°C**.

**Sample**	**Reaction rate 0°C (mol CO g Au^−1^s^−1^ × 10^4^)**	**Reaction rate 25°C (mol CO g Au^−1^s^−1^ × 10^4^)**	**TOF 0°C (s^−1^)**	**TOF 25°C (s^−1^)**	**Au particle size (nm)**	**Au dispersion**
Au/Ce/Al	4.37	6.86	1.33	2.10	4	0.32
Au/Fe/Al	3.28	5.08	1.00	1.55	4	0.32
Au/CeFe0.5/Al	6.45	7.26	1.97	2.21	4	0.32
Au/CeFe1/Al	5.69	5.74	1.74	1.76	4	0.32
Au/CeFe1.5/Al	4.70	6.35	1.43	1.94	4	0.32
Au/CeFe2/Al	6.12	6.12	1.87	1.87	4	0.32
Au/CeFe3/Al	3.66	6.54	1.08	1.99	4	0.32

In the case of the ternary solids, very good activity has been observed being the Au/Ce/Al system better than the Au/Fe/Al. At 25°C the results show that the Au/Fe/Al sample exhibits superior rate than a similar Au/Fe_2_O_3_ catalysts measured in comparable conditions reported in reference Kung et al. (2007) (5.08 × 10^−4^ vs. 3.4 × 10^−4^). In addition, the TOFs values of practically all the samples are higher than that calculated TOFs for similar Au/FeO_x_/CeO_2_-Al_2_O_3_ catalysts measured in the same conditions reported in Reina et al. (2012).

For the quaternary systems, an improvement of the activity is observed. However, a clear trend with the iron oxide loading cannot be established, indicating that the rate of the CO oxidation is governed mainly by the presence of gold and that a similar gold particle size results in a similar reaction rates. Nevertheless, the quaternary solids, exhibited better ability to abate CO compared to the ternary solids (Au/Ce/Al or Au/Fe/Al), being Au/CeFe2/Al the system that totally eliminate CO at lowest temperature (−32°C). It was reported, that the support could influence the activity indirectly either by increasing the number of low coordinated Au atoms (Janssens et al., 2007) or by the presence of the contact Ce-O-Fe surface due to the solid solution formation, at which interface a lowering of the energy barrier for the oxygen migration occurs (Trovarelli, 1999). Ce-Fe contact evidenced by our XPS and UV-Vis data resulted to be critical for a high performance in the CO oxidation. In this sense, Au/CeFe3/Al was the less active within the Ce-Fe mixed systems, meanwhile Au/CeFe2/Al was the most active. The later agrees with the information extracted on the UV-Vis data where Ce-Fe strongest interaction was achieved for the Au/CeFe2/Al whilst for the Au/CeFe3/Al solid iron oxide segregation was intended. Furthermore, the higher capacity of the Ce-Fe mixed catalysts to abate CO is also related to the improved redox properties demonstrated by the TPR and OSC experiments. Gold supported on iron doped-cerium oxide has a larger amount of labile reducible oxygen, which generally leads to more active catalyst in the oxidation reactions.

## Conclusions

A series of highly effective CO oxidation catalysts has been developed in the present paper. The materials are able to completely eliminate CO even at sub-ambient temperatures reaching full conversion of CO at (−32°C) in the best situation. XPS data points that the catalyst surface is enriched in cerium and iron oxide compared to the bulk and also evidences that the interaction between metallic gold particles and the support depends on the support composition. The modification of the ceria electronic properties by the inclusion of iron oxide has been demonstrated by the UV-vis experiments. Enhanced redox properties and oxygen mobility are achieved when iron is employed as a ceria dopant leading to promising materials for CO oxidation reactions with potential environmental applications.

As a final remark it should be underlined the viability of the systems based on inert support as a matrix for the homogeneous rare earth doped metal oxide catalytically active layer.

### Conflict of interest statement

The authors declare that the research was conducted in the absence of any commercial or financial relationships that could be construed as a potential conflict of interest.
